# Editorial: The role of immunometabolism in autoimmune mediated and autoinflammatory disorders

**DOI:** 10.3389/fimmu.2022.969939

**Published:** 2022-07-15

**Authors:** Valentina Pucino, Monica Guma

**Affiliations:** ^1^ Kennedy Institute of Rheumatology, University of Oxford, Oxford, United Kingdom; ^2^ Institute of Inflammation and Ageing, University of Birmingham, Birmingham, United Kingdom; ^3^ Division of Rheumatology, Allergy and Immunology, University of California, San Diego, San Diego, CA, United States

**Keywords:** autoimmune disease, immunometabolism, cell metabolism, inflammation, bioenergetic

Over the last years, cell metabolism has become one of the most exciting areas of investigation in the field of immuno-rheumatology. Convincing evidence has revealed that metabolic pathways closely regulate cell activities and immune as well as stromal cells adopt distinct metabolic programs to sustain their function in order to cope with environmental demands. It has been shown that dysregulated cell metabolism contributes to the development of several autoimmune diseases such as systemic lupus erythematosus (SLE), rheumatoid arthritis (RA), and multiple sclerosis (MS) where current treatment options are effective only in some patients. Predictive biomarkers of prognosis and therapeutic response remain inadequate from a clinical perspective. Thus, new therapeutic options as well as disease predictors are needed. Our aim in assembling this Research Topic is to highlight the current understanding of the role of cell metabolism in regulating immune responses in health and in autoimmunity with the purpose to find opportunities for clinical translation.

Qiu et al., focus on metabolic abnormalities of T cells from RA patients. They discuss how metabolic dysregulation is present in the naïve population and sustained in tissue-residing memory T cells, placing metabolic dysregulation upstream of the joint. Similar to RA T cells, type 1 diabetes (T1D) CD4+ T cells exhibit a pro-inflammatory phenotype which is accompanied by an enhanced glycolytic metabolism (Martins et al.) Martins et al., showed that targeting glycolysis with the use of the small molecule PFK15, a competitive inhibitor of the rate limiting glycolysis enzyme 6-phosphofructo-2-kinase/fructose-2,6- biphosphatase 3 (PFKFB3) was able to dampen inflammation *in vitro* and *in vivo*, in an adoptive transfer model of T1D.

Similar to T cells, synovial like fibroblasts (FLS) also show metabolic dysregulation in autoimmune arthritis. O’Brien et al. show that janus kinase (JAK)-signal transducer and activator of transcription (STAT) signalling mediate a complex interplay between inflammation and cellular metabolism in psoriatic arthritis (PsA). The inhibition of this pathway with JAK inhibitor shows effective suppression of inflammatory mechanisms that drive pathogenic functions of PsA FLS. Similarly Falconer et al. found that RA FLS display an impairment of mitochondrial function which alter their capability to resolve inflammation. The role of mitochondria in the pathogenesis of RA is further discussed by Clayton et al. who illustrate the role of mitochondria in the pathogenesis of RA and how current and future therapeutic strategies can function through modulation of mitochondrial activity.

Targeting glygolytic enzymes has also revealed to be a powerful tool to reduce inflammation in RA and in a collagen induced arthrtits mouse model as outlined by Zuo et al. and Wang et al. respectively. Continuing on the field of arthritis, Tripolino et al. discuss the cause-effect relationship between arthritis and metabolic abnormalities with a focus on insulin signaling. They also offer their view on the effect of glucose-lowering agents on arthritis. On the same topic, Jutley et al., assess the relationship between an objective measure of systemic inflammation [C-reactive protein (CRP)] and both the serum and urinary metabolome in patients with newly presenting RA. These findings suggest that NMR spectroscopy is a valid tool for the identification of metabolic biomarkers in disease. New metabolic pathways are emerging as novel potential driver of inflammation in RA. In this vein, Zhao et al. speculate the possible role of ferroptosis in the pathogenesis of RA. Mormile et al. illustrate the multifaceted roles of formyl peptide receptors in promoting resolution or inflammation in RA. Moving toward other autoimmune diseases, De Luca et al. illustrate the link between IL1, metabolism, inflammasome activation and cardiovascular complications in systemic sclerosis. Nardone et al. discuss the relationship between gut microbiota and sarcopenia in inflammatory bowel disease. Robinson et al. highlight the latest understanding of the role of immunometabolism in SLE with particular focus on the role of abnormal mitochondrial function, lipid metabolism, and mammalian target of rapamycin (mTOR) signaling. Hwang et al. examined the impacts of changes associated with aging or metabolic abnormalities on populations of T and B cells and Sjogren’s disease severity. Peruzzotti-Jametti discuss how cell metabolism and mitochondrial function govern the function of chronic active microglia and macrophages in neuroinflammatory conditions. Finally, Lin et al. in their review discuss the role of reactive oxygen species (ROS) in regulating interactions between innate and adaptive immunity in autoimmunity. Raza and Clarke offer an overview of B cell metabolism and autophagy in health and disease.

In conclusion, these are exciting times for those who are investigating cell metabolism during homeostasis and in autoimmunity. Gaining deeper understanding of how cell metabolism and immune responses regulate each other will lead to new insights on disease mechanisms and, eventually, to the development of novel therapeutic options.

## Author contributions

All authors listed have made a substantial, direct, and intellectual contribution to the work and approved it for publication.

## Funding

VP is supported by the National Institute for Health Research (NIHR) Birmingham Biomedical Research Centre. This work was supported by the National Institutes of Health (R01AR073324 to MG).

## Conflict of interest

The authors declare that the research was conducted in the absence of any commercial or financial relationships that could be construed as a potential conflict of interest.

## Publisher’s note

All claims expressed in this article are solely those of the authors and do not necessarily represent those of their affiliated organizations, or those of the publisher, the editors and the reviewers. Any product that may be evaluated in this article, or claim that may be made by its manufacturer, is not guaranteed or endorsed by the publisher.

